# Group A *Streptococcus* Primary Peritonitis in Children, New Zealand

**DOI:** 10.3201/eid2911.230211

**Published:** 2023-11

**Authors:** Amanda Taylor, Brodie M. Elliott, John Atkinson, Sally Roberts, Lesley Voss, Emma J. Best, Rachel Webb

**Affiliations:** Starship Children’s Hospital, Auckland, New Zealand (A. Taylor, B.M. Elliott, J. Atkinson, L. Voss, E.J. Best, R. Webb);; The University of Auckland, Auckland (A. Taylor, E.J. Best, R. Webb);; LabPLUS, Auckland City Hospital, Auckland (S. Roberts);; Kidz First Hospital, Counties Manukau, Auckland (R. Webb)

**Keywords:** streptococci, bacteria, group A Streptococcus, *Streptococcus pyogenes*, peritonitis, New Zealand

## Abstract

Group A *Streptococcus* (GAS) primary peritonitis is a rare cause of pediatric acute abdomen (sudden onset of severe abdominal pain); only 26 pediatric cases have been reported in the English language literature since 1980. We discuss 20 additional cases of pediatric primary peritonitis caused by GAS among patients at Starship Children’s Hospital, Auckland, New Zealand, during 2010–2022. We compare identified cases of GAS primary peritonitis to cases described in the existing pediatric literature. As rates of rates of invasive GAS increase globally, clinicians should be aware of this cause of unexplained pediatric acute abdomen.

*Streptococcus pyogenes*, or group A *Streptococcus* (GAS), causes a wide spectrum of disease ranging from superficial skin infection and pharyngitis to invasive infections, such as sepsis, empyema, necrotizing fasciitis, meningitis, osteomyelitis, and septic arthritis (*1*). GAS is responsible for the toxin-mediated complications of scarlet fever and streptococcal toxic shock syndrome, as well as the postinfectious sequelae rheumatic fever and poststreptococcal glomerulonephritis (*2*).

Primary peritonitis is defined as a bacterial infection within the peritoneal cavity in the absence of ascites or an intraabdominal source of infection, such as appendicitis (*3*,*4*). Primary peritonitis is an uncommon manifestation of invasive GAS (iGAS) disease; a 2016 report found it accounted for 4.6% of children with iGAS in Finland (*5*). Pediatric primary peritonitis itself is rare, reportedly accounting for 1%–3% of children experiencing acute abdomen (sudden onset of severe abdominal pain), and is most commonly caused by *S. pneumoniae,* gram-negative organisms, and staphylococcal species (*3*).

In late 2022, the United Kingdom, United States, Australia, and several countries in Europe reported unexpectedly high rates of iGAS and scarlet fever, particularly in children <10 years of age (*6*–*10*). Rates of iGAS in the United Kingdom during this period are reported to be higher than in the years before the COVID-19 pandemic (2017–2019) and substantially higher than those reported during 2020–2021 (*11*). With increased GAS circulation, media coverage, and heightened community awareness, clinicians globally should be aware of the vast spectrum of invasive disease caused by GAS, including primary peritonitis.

In New Zealand, iGAS disease is not notifiable to Public Health authorities (*12*). Surveillance relies on individual laboratories sending clinically relevant iGAS isolates to the Institute of Environmental Science and Research (ESR) for typing (*13*). The most recent ESR report (2016) describes an iGAS incidence of 9.0/100,000 population in New Zealand; rates were inequitably high among New Zealand Māori (23.5/100,000 population) and Pacific peoples (73.9/100,000 population) compared with rates among persons of European or other ethnicities (4.3/100,000 population) (*13*). Incidence of iGAS is highest in children <1 year of age (*13*). Although ESR laboratory surveillance provides some information on the burden of iGAS in New Zealand, its passive nature suggests that reported rates are likely to be an underestimate.

Consistent with the high burden of iGAS disease in children in New Zealand, clinicians at Starship Children’s Hospital in Auckland have observed that GAS is a major cause of primary peritonitis in the pediatric population. We evaluated the incidence, clinical features, and management of children admitted to this tertiary pediatric center with GAS primary peritonitis during January 1, 2010–June 30, 2022, to provide a better understanding of the clinical features of GAS primary peritonitis in a contemporary setting with a high burden of iGAS disease. In addition, we reviewed and compared identified cases with reports in the English language literature.

## Methods

We performed a retrospective observational study of children admitted to Starship Children’s Hospital with GAS primary peritonitis during January 1, 2010–June 30, 2022. Starship Children’s Hospital is a tertiary hospital with a surgical department that primarily serves Auckland and northern New Zealand. It contains the country’s only pediatric intensive care unit (PICU), and complex cases from across New Zealand are frequently transferred to the facility for surgical management.

We included children <15 years of age who had primary peritonitis, defined as bacterial infection within the peritoneal cavity in the absence of ascites or an intraabdominal source of infection either confirmed on abdominal imaging (typically free intraabdominal fluid and peritoneal enhancement with small bowel dilation and bowel wall thickening) (*14*) or operative findings (free fluid in the abdomen without intraabdominal or gynecological pathology) (*3*); and from whom GAS had been isolated from culture or molecular methods from blood culture, peritoneal fluid, peritoneal tissue, or intraoperative peritoneal swab samples (*3*,*4*). We excluded children with peritonitis secondary to preexisting ascites or an indwelling peritoneal dialysis catheter or device; children in whom an intraabdominal source of infection, such as a perforated viscus, acute appendicitis, or gynecological pathology, had been identified; or in whom another bacterial cause of primary peritonitis had been identified, by culture or molecular method, from any of blood culture, peritoneal fluid, peritoneal tissue, or intraoperative peritoneal swab sample.

A.T. reviewed electronic notes, imaging, operative findings, and microbiology of all possible cases to determine whether persons met the study criteria. A multidisciplinary team of the remaining authors then discussed possible cases; the team consisted of pediatric surgeons, pediatric infectious disease specialists, and a clinical microbiologist.

We reviewed 4 data sources for case ascertainment. The first source was Pediatric Infectious Diseases (ID) daily handover lists from the Starship Pediatric ID service inpatient consultation service, which are Microsoft Word documents stored in a password-protected folder on the Starship hospital server. They are manually updated each day by the Starship Pediatric ID team with a summary of all active referrals, including patient diagnosis and relevant microbiology. We searched all handover lists during the study period were electronically searched for the terms “peritonitis,” “group A *Streptococcus*,” “GAS” and/or “*Streptococcus pyogenes.*” Second, we searched for codes from the International Classification of Diseases, 10th Revision (ICD-10), from discharge summaries of patients at Starship Children’s Hospital during the study period for primary diagnosis peritonitis or acute peritonitis (codes K65.-, P78.0, P78.1, N73.3, N73.4, N73.5, K35.2, K35.3, K57.0-, K57.2-, K57.4-, K57. 8-), with or without secondary group A streptococcus (codes B95.0, A40.0). Third, we requested national reference laboratory data from the New Zealand ESR for children <15 years of age with iGAS isolates sent from LabPLUS (Starship Hospital laboratory service) for *emm* typing. All laboratories in New Zealand are asked to send clinically relevant iGAS isolates to ESR for typing. Clinically relevant iGAS isolates are defined as GAS from a normally sterile body site (e.g., blood, cerebrospinal fluid, pleural fluid, peritoneal fluid, synovial fluid) consistent with the US Centers for Disease Control and Prevention definition of iGAS (*13*). Last, we reviewed LabPLUS microbiology data for children with GAS culture–positive or molecular test–positive peritoneal or abdominal aspirates, tissue, or peritoneal swab samples and GAS-positive blood cultures.

We subsequently conducted a review of the English literature from 1980 onward using PubMed and the search terms “streptococcus pyogenes” [MeSH] OR “group A streptococc*” OR “streptococcal infection* OR “invasive GAS” OR “invasive group A streptococc*” AND primary peritonitis [MeSH]. We identified additional cases by reviewing references and citations of articles identified from the initial PubMed search. We excluded cases in which a primary etiology of peritonitis (e.g., peritoneal dialysis, gynecological or abdominal pathology) had been identified. 

This study received Auckland Health Research Ethics Committee approval (AH24823). We performed data collection and descriptive analyses using Microsoft Excel.

## Results

We identified 253 possible cases at the hospital during the study period. Of those, 20 cases met the study inclusion criteria for GAS primary peritonitis (Appendix Table). Only 2/20 (10%) were identified from all 4 data sources and 5/20 (20%) were notified to ESR by LabPLUS. We compared patient demographics and clinical features to features described in previous case reports (Table).

**Table:** Comparison of characteristics of cases of pediatric GAS primary peritonitis at Starship Children’s Hospital, New Zealand, 2010–2022, to previously described cases*

Pediatric GAS primary peritonitis	Cases from literature review, 1980–2022, n = 26 [*3*,*4*,*14–30*]	Cases from Starship Hospital, Jan 1, 2010–Jun 30, 2022, n = 20
Median age (range)	7 y (2 wk–16 y)	2 y (3 wk–13 y)
Sex		
F	16/19 (84)	11/20 (55)
M	3/19 (16)	9/20 (45)
Preceding throat or skin infection		
Pharyngitis	4/18 (22)	1/20 (5)
Skin	3/18 (17)	5/20 (25)
STSS	7/18 (39)	6/20 (30)
Microbiology		
Peritoneal fluid or tissue	25/26 (96)	17/20 (85)
Blood culture	5/26 (19)	9/20 (45)
Other	1/26†	4/20 (20)‡
Molecular method (16S)	3/25 (12)	0/20
Culture	22/25 (88)	19/20 (95)
Surgery		,
Laparoscopy or laparotomy	21/25 (84%)§	19/20 (95)
VATS only	NA	1/20 (5)
Median antibiotic duration (range), d	14 (10–61)	21 (14–42)
Clindamycin treatment	4/14 (29)	4/20 (20)
IVIG treatment	3/14 (21)	1/20 (5)
Median length of hospital stay (range), d	10 (4–47)	13 (5–31)
Deaths	0/19	0/20
*emm* types	3.1 (n = 1)	88 (n = 1), 65/69 (n = 1), 114 (n = 2), 118 (n = 1)§
M-type	M3 (n = 1), M81 (n = 1), M2 (n = 1)	NA

*Values are no. (%) except as indicated. IVIG, intravenous immunoglobulin; NA, not available; STSS, streptococcal toxic shock syndrome; VATS, video-assisted thoracoscopic surgery. †From pleural aspirate. ‡From skin swab samples; 3/4 GAS-positive skin swabs also had a positive blood or intraoperative peritoneal culture. §5/20 isolates were sent for *emm* typing.

Ethnicity was recorded as New Zealand Māori for 3/20 (15%) children and Pacific peoples for 8/20 (40%) children. Comorbidities were present in 3 children: 1 child was born at 29 weeks’ gestation with hypoxic ischemic encephalopathy, 1 had suspected immunodeficiency, and 1 child had a periodic fever syndrome.

Median symptom duration before admission was 3.5 days (range 1–8 days). We reviewed clinical notes to identify possible skin or pharyngeal sources. A possible skin source was identified in 5/20 (25%) children; sources were a foot wound with cellulitis, a leg wound with cellulitis, vaginal erythema and discharge, groin cellulitis, and scrotal erythema with discharge. Skin swab samples were culture-positive for GAS in 4/5 children with possible skin sources. One child was documented as having pharyngitis the week before admission, although no throat swab sample was collected. Although the presence of skin or pharyngeal infection in household contacts was infrequently documented, 1 child’s father had confirmed GAS pharyngitis the week before the child’s hospitalization.

Of the 22 cases identified as primary peritonitis on ICD-10 codes, 13/22 had positive microbiology. GAS was cultured from 11/22 (50%). One case each of *S. pneumoniae* and *S. anginosus* primary peritonitis was identified.

A total of 19/20 cases of GAS primary peritonitis were culture-positive for GAS from blood or intraoperative peritoneal samples. In 3/19 cases, GAS was cultured from blood or peritoneal samples at the original hospital before the patient was transferred to Starship Hospital. In 7/20 cases, both blood culture and peritoneal culture were GAS-positive (on tissue, aspirate, or intraoperative swab sample). Two of 20 cases were blood culture–positive and peritoneal culture–negative. Half (10/20) were peritoneal culture–positive and blood culture–negative. No patients were identified by molecular methods.

We included 1 patient despite negative blood and peritoneal cultures because intraoperative and imaging findings were consistent with primary peritonitis and because GAS was cultured from a swab sample of vaginal discharge taken at admission. That patient had 2 intraoperative peritoneal swab samples (obtained after antimicrobial treatment) that were culture-negative but demonstrated gram-positive cocci. No molecular method could be performed on the swabs. It was the multidisciplinary team’s consensus opinion that this patient should be included as a probable case of GAS primary peritonitis.

One quarter (5/20) of iGAS isolates from this series were sent to ESR from LabPlus for typing and surveillance. In total, primary peritonitis represented 4.6% (5/108) of pediatric iGAS isolates sent to ESR from LabPLUS during the study period.

US Centers for Disease Control and Prevention criteria for streptococcal toxic shock syndrome (STSS) were met in 6/20 cases (30%), and 10/20 (50%) patients were admitted to the PICU; 5/10 (50%) patients required ventilation and 6/10 (60%) required inotropes (*9*). A total of 4/20 (20%) patients were given adjunctive clindamycin, and 1/20 received intravenous immunoglobulin. Of those who received clindamycin, 2 patients had STSS, 1 had presumed β-lactam–induced neutropenia, and 1 had clindamycin added to their existing treatment for evolving empyema. The average duration of total antibiotic treatment in our series was 21 days (range 14–42 days), and the average hospital stay was 13 days (range 5–31 days). No deaths occurred within 90 days of hospital admission. One patient was readmitted to the hospital because of intolerance of oral antibiotics.

## Discussion

GAS primary peritonitis is a rare but noteworthy cause of pediatric acute abdomen; only 26 pediatric cases have been reported in the English literature since 1980 (Table) (*3*,*5*,*14*–*30*). We describe a large single-center pediatric cohort of 20 cases in patients admitted to Starship Children’s Hospital, New Zealand, during 2010–2022. This study contributes to existing knowledge of the clinical manifestations, treatment, and trajectory of children with GAS primary peritonitis. In the context of increasing rates of iGAS globally, our case series should increase awareness among clinicians of this manifestation of iGAS disease.

In our series, GAS was the most common pathogen isolated from children with an ICD-10 discharge code of primary or acute peritonitis. This finding contrasts with previous reports that found more common pediatric primary peritonitis causes were *S. pneumoniae*, staphylococci, and gram-negative organisms (*3*). Although this retrospective, observational study did not aim to assess the causative pathogens of all primary peritonitis cases, key pathogens could have changed over time; this possibility should be the focus of further study. Our findings could be explained by the high burden of iGAS in New Zealand children, particularly among Māori and Pacific peoples, who were overrepresented in our case series (accounting for 55% of cases).

Postulated causes of GAS primary peritonitis include hematogenous spread from the skin or respiratory tract, ascending infection from the female genital tract, and gastrointestinal translocation (*4*,*30*). Although GAS is known to colonize the throat, skin, and female genital tract, it is not typically present in intestinal flora (*30*). Among previously reported pediatric cases, a possible skin or throat source was found in 50% of cases (*14*). In our cohort, a skin or throat source was identified in a similar proportion of cases (30%). Because of the retrospective nature of our study, clinical records were not always complete. Future studies would benefit from standardized, prospective collection of cases detailing recent pharyngeal, skin, or genitourinary infections (*1*,*3*).

Previous reports have suggested that girls are disproportionally affected by GAS primary peritonitis because of ascending genitourinary infection; in 84% (16/19) of previous pediatric case reports where sex was given, the child was a girl (*30*). This effect was not seen in our cohort, where the median age was younger than that previously reported and 55% of case-patients were girls, suggesting that this route might be less common in younger children (*30*).

As in previous reports, symptoms of children in our cohort were abdominal pain, nausea, vomiting, and diarrhea. Several cases were initially diagnosed as viral gastroenteritis before deteriorating into peritonism and sepsis (*3*,*14*). Of the children in our cohort, 30% met STSS criteria, which is comparable with previous case reports that found an overall STSS incidence of 39% (*5*,*14*,*15*).

Although adult clinical practice guidelines advise nonoperative management, particularly in persons with underlying ascites, previous case reports found that most (83%) pediatric patients undergo surgery to rule out secondary peritonitis (*14*,*30*). This practice was also demonstrated in our series; 95% of patients underwent laparoscopy or laparotomy. Surgical management might aid source control, particularly in patients with STSS where reduction of bacterial load plays a major role (*4*,*14*,*31*). Operative management has also been hypothesized to decrease the duration of illness: a 2020 case report describes a child who was managed nonoperatively and in whom a multi-loculated abscess developed that required multiple drains and 61 days of antibiotics (*30*).

The median total duration of antibiotic treatment in our cohort was 21 days (range 14–42 days), which is 1 week longer than the median duration of 14 days (range 10–61) seen in previous case reports. The additional benefit of clindamycin or intravenous immunoglobulin in the management of GAS primary peritonitis remains unclear; however, those adjunct treatments might have a role in patients with STSS.

Observational case reports alone make it difficult to comment on the importance of operative management or the optimal duration of antibiotic treatment for GAS primary peritonitis; however, despite substantial rates of severe illness in our series (10 cases [50%] required PICU admission), no deaths occurred, and only 1 patient required readmission to the hospital. Furthermore, no deaths have been reported in any pediatric case report of GAS primary peritonitis since the 1980s (*14*). As such, shorter durations of antibiotics may be reasonable for patients with less severe clinical manifestation, particularly when early surgical source control is achieved.

Although this report describes iGAS infection in a population with an identified high burden of GAS infection, as reports emerge from Europe, the United Kingdom, and the United States describing an increase in iGAS and scarlet fever, pediatricians should be aware of this cause of acute abdomen (*6*). Identifying iGAS promptly enables antibiotic treatment to be rationalized to penicillin and might enable STSS to be recognized earlier. The international rise in iGAS cases might also provide an opportunity to evaluate the best treatment approach for GAS primary peritonitis and how best to manage close contacts. In New Zealand, no guidelines exist around routine assessment of household contacts of persons with iGAS. The risk for secondary iGAS disease in household contacts is estimated to be 2,000 times higher than the general population risk, substantially higher than that reported for meningococcal disease (500–800 times), which is a notifiable condition in New Zealand (*32*). Therefore, identifying highly transmissible iGAS should prompt timely review, collection of throat swab specimens, and consideration of chemoprophylaxis for high-risk household contacts through existing public health communicable disease control services for meningococcal disease and other high-priority close contact infectious diseases (*32*).

During the study period, GAS primary peritonitis accounted for 4.2% of pediatric iGAS isolates sent to the national reference laboratory from Starship Children’s Hospital for *emm*-typing and surveillance. Although this is comparable with a 2016 national surveillance study of pediatric iGAS disease in Finland, which reported a primary peritonitis incidence of 4.6% (7/151) (*5*), in our series only 5/20 patients with GAS primary peritonitis had their isolates sent for *emm*-typing and surveillance. Starship Hospital is a tertiary referral center, and 3/19 iGAS isolates were cultured at other hospitals in New Zealand. Those isolates could have been sent to ESR by those hospital laboratories, but our observation that only a small number of culture-positive samples at our hospital were sent to ESR suggests that passive surveillance is likely to underestimate the true burden of iGAS in New Zealand. This case series highlights the difficulties of case ascertainment, disease surveillance, and the ability to monitor circulating *emm*-types when a disease is not monitored through prospective national surveillance. In a time of increasing global rates of iGAS disease, the results of this study strengthen calls for enhanced, prospective, national iGAS surveillance (*1*,*6*,*12*,*32*). Although this cohort is small, strengths include robust case ascertainment from multiple data sources and comprehensive classification of cases through detailed review by a multidisciplinary team of clinicians.

In conclusion, GAS primary peritonitis is an uncommon cause of iGAS disease in children but can cause severe illness. This contemporary case series describes 20 children over a 12.5-year period, 30% of whom met the criteria for STSS and 50% of whom required PICU admission. Our findings reflect the substantial burden of GAS disease in New Zealand. In a time of increasing iGAS rates globally, GAS primary peritonitis should be considered in children experiencing unexplained acute abdomen.

Appendix Additional information about group A *Streptococcus* primary peritonitis in children, New Zealand.
