# Supplementary material for: Group A Streptococcus Primary Peritonitis in Children, New Zealand
Source: Emerg Infect Dis. 2023 Nov;29(11):2203–9. doi: 10.3201/eid2911.230211 (PMC10617357; doi:10.3201/eid2911.230211)
Supplement: Appendix — Additional information about group A Streptococcus primary peritonitis in children, New Zealand. [file 23-0211-Techapp-s1.pdf]

Article DOI: https://doi.org/10.3201/eid2911.230211

*EID cannot ensure accessibility for supplementary materials supplied by authors. Readers who have difficulty accessing supplementary content should contact the authors for assistance.*

# Group A *Streptococcus* Primary Peritonitis in Children, New Zealand

## Appendix

**Appendix Table.** Cases of GAS primary peritonitis admitted to Starship Children's Hospital, New Zealand, January 1, 2010–June 30, 2022, n = 20*

| Year | Sex/age | Ethnicity | GAS culture site | Possible skin or pharyngeal source | PICU | Ventilated | Inotropes | Clindamycin or IVIG | STSS | Laparoscopy or laparotomy | Total antibiotic duration (days) | LOS (days) | *emm* type |
|---|---|---|---|---|---|---|---|---|---|---|---|---|---|
| 2010 | M/10 mo | NZ European | Blood, peritoneal swab | No | Yes | No | No | No | No | Yes | 28 | 11 | 65/69 |
| 2010 | F/17 mo | NZ European | Blood, peritoneal aspirate | No | No | No | No | No | No | Yes | 14 | 10 | X |
| 2010 | M/5 y | Other European | Peritoneal aspirate, peritoneal swab | Skin | Yes | Yes | Yes | Clindamycin, IVIG | Yes | Yes | 21 | 9 | X |
| 2010 | F/11 y | NZ European | Peritoneal swab | Pharyngitis, not swabbed | No | No | No | No | No | Yes | 16 | 9 | X |
| 2011 | F/4 mo | Pacific | Vaginal swab, peritoneal swab Gram positive cocci† | Skin | No | No | No | No | No | Yes | 14 | 9 | X |
| 2011 | F/6 y | NZ Māori | Blood, peritoneal aspirate, peritoneal swab | No | Yes | Yes | Yes | No | Yes | Yes | 28 | 28 | X |
| 2011 | F/2 y | Pacific | Blood, peritoneal aspirate, peritoneal swab | No | Yes | No | No | Clindamycin | No | Yes | 42 | 31 | 118 |

| Year | Sex/age | Ethnicity | GAS culture site | Possible skin or pharyngeal source | PICU | Ventilated | Inotropes | Clindamycin or IVIG | STSS | Laparoscopy or laparotomy | Total antibiotic duration (days) | LOS (days) | *emm* type |
|---|---|---|---|---|---|---|---|---|---|---|---|---|---|
| 2013 | M/14 mo | Pacific | Blood, peritoneal aspirate | No | Yes | Yes | Yes | No | Yes | Yes | 21 | 21 | X |
| 2014 | M/3 wks | NZ Māori | Blood, Peritoneal aspirate | No | Yes | Yes | Yes | No | No | Yes | 28 | 28 | X |
| 2015 | M/8 mo | Pacific | Peritoneal aspirate, peritoneal tissue, peritoneal swab, skin swab | Skin | Yes | No | Yes | No | No | Yes | 17 | 7 | X |
| 2016 | F/9 mo | Pacific | Peritoneal aspirate, peritoneal swab | Skin, not swabbed | No | No | No | No | Yes | Yes | 14 | 9 | X |
| 2016 | F/2 y | Pacific | Blood, skin swab | Skin | Yes | No | No | No | No | Yes | 28 | 23 | X |
| 2017 | F/5 mo | NZ European | Blood, peritoneal aspirate, peritoneal swab | No | Yes | Yes | Yes | No | Yes | Yes | 21 | 16 | X |
| 2019 | F/2 y | Pacific | Blood, peritoneal tissue, peritoneal swab | No | No | No | No | Clindamycin | No | Yes | 28 | 28 | X |
| 2019 | M/3 y | NZ European | Peritoneal tissue | No | No | No | No | No | No | Yes | 14 | 5 | X |
| 2019 | F/13 y | NZ European | Peritoneal tissue | No | No | No | No | No | No | Yes | 21 | 12 | X |
| 2021 | M/3 y | NZ Māori | Peritoneal aspirate | No | No | No | No | No | No | Yes | 21 | 14 | 114 |
| 2021 | M/4 y | NZ European | Peritoneal tissue | No | No | No | No | No | No | Yes | 21 | 9 | X |
| 2021 | M/5 y | NZ European | Blood | No | No | No | No | No | No | No | 21 | 15 | 114 |
| 2022 | F/7 mo | Pacific | Peritoneal aspirate, peritoneal swab | No | Yes | No | No | Clindamycin | Yes | Yes | 21 | 14 | 88 |

*GAS, group A *Streptococcus*; IVIG, intravenous immunoglobulin; LOS, length of stay; NZ, New Zealand; PICU, pediatric intensive care unit; STSS, streptococcal toxic shock syndrome.
†Case included following multidisciplinary team discussion and consensus opinion. Vaginal erythema and discharge at presentation was swabbed and culture positive for GAS. One blood culture negative. Only intraoperative peritoneal sample collected were swabs which was not suitable for molecular methods but had gram-positive cocci seen on Gram stain.
